# Effects of smoking status, history and intensity on heart rate variability in the general population: The CHRIS study

**DOI:** 10.1371/journal.pone.0215053

**Published:** 2019-04-09

**Authors:** Federico Murgia, Roberto Melotti, Luisa Foco, Martin Gögele, Viviana Meraviglia, Benedetta Motta, Alexander Steger, Michael Toifl, Daniel Sinnecker, Alexander Müller, Giampiero Merati, Georg Schmidt, Alessandra Rossini, Peter P. Pramstaller, Cristian Pattaro

**Affiliations:** 1 Institute for Biomedicine, Eurac Research, Affiliated Institute of the University of Lübeck, Bolzano, Italy; 2 Medizinische Klinik und Poliklinik, Klinikum rechts der Isar, Technische Universität München, Munich, Germany; 3 DZHK (German Centre for Cardiovascular Research), Partner Site Munich Heart Alliance, Munich, Germany; 4 Dipartimento di Scienze Biomediche per la Salute, Università degli Studi di Milano,Milano, Italy; 5 Centro di Medicina dello Sport, Fondazione Don C. Gnocchi, Milano, Italy; 6 Department of Neurology, General Central Hospital, Bolzano, Italy; 7 Department of Neurology, University of Lübeck, Lübeck, Germany; University of Tampere, FINLAND

## Abstract

**Background:**

Heart rate variability (HRV) reflects the autonomous nervous system modulation on heart rate and is associated with several pathologies, including cardiac mortality. While mechanistic studies show that smoking is associated with lower HRV, population-based studies present conflicting results.

**Methods:**

We assessed the mutual effects of active smoking status, cumulative smoking history, and current smoking intensity, on HRV among 4751 adults from the Cooperative Health Research In South Tyrol (CHRIS) study. The HRV metrics standard deviation of normal-to-normal (NN) inter-beat intervals (SDNN), square root of the mean squared differences of consecutive NN intervals (RMSSD), total power (TP), low (LF) and high frequency (HF) power, and their ratio (LF/HF), were derived from 20-minute electrocardiograms. Smoking status, pack-years (PY), and tobacco grams/day from standardized questionnaires were the main exposures. We fitted linear mixed models to account for relatedness, non-linearity, and moderating effects, and including fractional polynomials.

**Results:**

Past smokers had higher HRV levels than never smokers, independently of PY. The association of HRV with current smoking became apparent when accounting for the interaction between smoking status and PY. In current smokers, but not in past smokers, we observed HRV reductions between 2.0% (SDNN) and 4.9% (TP) every 5 PY increase. Furthermore, current smokers were characterized by dose-response reductions of 9.8% (SDNN), 8.9% (RMSSD), 20.1% (TP), 17.7% (LF), and 19.1% (HF), respectively, every 10 grams/day of smoked tobacco, independently of common cardiometabolic conditions and HRV-modifying drugs. The LF/HF ratio was not associated with smoking status, history, or intensity.

**Conclusions:**

Smoking cessation was associated with higher HRV levels. In current smokers, heavier smoking intensity appears gradually detrimental on HRV, corroborating previous evidence. By affecting both the sympathetic and parasympathetic nervous system indexes, but not the LF/HF balance, smoking intensity seems to exert a systemic dysautonomic effect.

## Background

Heart rate variability (HRV) is defined as the temporal variation between consecutive heartbeat intervals [1]. It mainly reflects the dynamic and adaptive modulation of the rhythmicity of the sinoatrial node by stimuli from the autonomous nervous system (ANS). The HRV is usually estimated through metrics extracted from electrocardiogram (ECG) traces. Such metrics reflect time and frequency domains of the heart activity [2] and return complementary information on the parasympathetic activity and on the sympathetic/parasympathetic balance of the ANS [2].

HRV assessment has gained interest as a non-invasive tool to obtain relevant information on cardiac autonomic regulation [1]. HRV imbalance has been proposed as a prognostic marker in several diseases, including neurological and psychiatric disorders [3; 4] and cancer [5]. A reduced HRV is associated with heart failure [6], diabetes [7], and all major cardiovascular risk factors [8] and is a predictor of cardiac events [9]. In the elderly, both decreased and increased HRV may predict cardiac mortality [10]. Several physiological factors may affect HRV in the general population. HRV declines with age [11], but remains persistently high in the healthy elderly [12]. Lifestyle is associated with HRV. For instance, weight loss following diet and exercise is associated with increased parasympathetic and decreased sympathetic activity, while weight gain having the opposite effect [13]. Regular physical exercise at low-to-moderate intensity is also associated with better cardiac autonomic nervous function [14]. In the last decades in particular, several studies explored the relationship between HRV and several smoking habits. Active tobacco smoking may have both chronic and acute effects on HRV [15,16]. However, evidence from population-based epidemiological studies is scant. A diverse range of study reports return either small associations [17] or a lack thereof [18; 19; 20] between smoking and HRV indexes. Active smoking behavior is rarely considered according to multiple constructs, including the three major aspects of status, history and intensity in any single study. In this work, we present the results of a detailed analysis, applied to a large sample from the general adult population of a rural Alpine environment. Using HRV metrics over both time- and frequency-domains, we show how different aspects of active smoking behavior relate to HRV. We disentangle the effects of status, cumulative history, and current intensity, in a structured analytical framework and accounting for major cardiometabolic risk factors.

## Methods

### Study population

The Cooperative Health Research In South Tyrol (CHRIS) study is a population-based study being carried out in South Tyrol (Italy) to investigate the basis of chronic conditions associated with human ageing. The study has been extensively described elsewhere [21, 22]. All participants underwent blood drawing, urine collection, anthropometry analysis, and clinical assessments in the early morning following overnight fasting. Participants had been asked not to smoke or drink alcohol or caffeine-rich beverages for at least 3 hours prior to the visit. Detailed medical history was reconstructed through both interviewer- and self-administered questionnaires. Participants were asked to report genealogical information about parents and grandparents, allowing the reconstruction of up-to-five generation pedigrees [22].

#### Heart rate variability (HRV)

Study participants underwent a 20-minute ECG in supine resting position, on a 12-lead PC-ECG-System Custom 200 machine, following a standard protocol as suggested by the Task Force of the European Society of Cardiology and the North American Society of Pacing and Electrophysiology [1]. ECG raw data were processed using the libRASCH software program [23]. QRS-annotations were visually validated and, if necessary, manually corrected by experienced physicians. In this study, we considered the following time-domain HRV metrics: the standard deviation of the normal-to-normal interbeat intervals (SDNN) and the square root of the mean squared differences of consecutive inter beat intervals (RMSSD). Power spectral analysis was used to obtain frequency-domain HRV metrics, namely total power (TP) (≤0.40 Hz), low frequency (LF) power (0.04–0.15 Hz), and high frequency (HF) power (0.15–0.40 Hz). Additionally, we estimated the LF/HF ratio.

#### Smoking behavior

Interviewer administered questionnaires adapted from the Cooperative Health Research in the Augsburg Region (KORA) study [24] and the European Community Respiratory Health Survey (ECRHS) [25] were harmonized to derive multiple measures of tobacco smoking behavior. Ever smokers reported the consumption of predefined amounts of tobacco or the regular consumption thereof in a lifetime, according to each questionnaire’s structure, including ever smoking cigarettes, cigars, and pipes, for a period of time or until present. Among ever smokers, anyone who reported smoking in the past month was considered a current smoker; otherwise, they were classified as past smokers. Twelve subjects who reported cessation in the last month were nonetheless classified as past smokers, in accordance to the KORA questionnaire structure. Never smokers were those, who never consumed the predefined amount of tobacco in their lifetime and were not currently smoking any amount.

Total tobacco grams were calculated by summing up the overall amount of tobacco of any type, including cigarettes, cigars, pipes, and cigarillos, typically smoked on a day. Published conversion rules were adopted to equalise different types of tobacco, with 1 cigarette equivalent to 1 gram, 1 cigar corresponding to 5 grams, 1 average size pipe tobacco to 2.85 grams, and 1 cigarillo to 3 grams [26]. Smoking intensity was operationalized as grams of total tobacco intake on a typical day. Among current smokers, smoking intensity allowed to isolate the acute effect of smoking at present consumption rates. Smoking intensity was also derived among ever smokers in order to calculate the amount of pack-years. Pack-years were obtained as the proportion equivalent to 20 grams of total tobacco (corresponding to one standard pack of cigarettes) typically smoked on a day times the years of reported smoking duration. The amount of pack-years represents a measure of cumulative smoking history.

#### General health conditions

The body-mass-index (BMI) was calculated as (weight in kg) / (height in m^2^). Diabetes was defined as self-reported doctor-diagnosed diabetes or use of glucose-lowering drugs, assessed via interviewer-administered questionnaire, or as a percentage of glycosylated haemoglobin (HbA1c) ≥6.5%. History of cardiovascular events was recorded via interviewer-administered questionnaire, by asking participants whether a doctor ever diagnosed any of the following conditions: coronary artery disease, myocardial infarction, angina pectoris, arrhythmic disease, myocarditis, cardiomyopathy, heart failure, or assuming cardiac medications (ATC code: C01). Systolic and diastolic blood pressure (SBP and DBP, respectively) were derived as the mean of three measurements taken every 2 minutes at the end of the 20-minute ECG, in supine position. Hypertension was defined as positive response to the question “has a doctor ever said that you have high blood pressure or hypertension?” or as having either a SBP ≥140 mmHg or a DBP ≥90 mmHg. Physical activity was classified as low, medium, or high, based on the total score derived from the short version of the self-administered International Physical Activity Questionnaire (IPAQ) [27]. We generated a categorical variable which classifies the use of medication into three groups: individuals who take no drugs; individuals who take at least one drug that potentially modifies HRV levels (HRV-modifying drugs); and individuals who take any other kind of drug except those that might modify HRV levels (other drugs). In the HRV-modifying drugs group we included anti-hypertensive medications (ATC codes: C02, C03, C04, C07, C08, and C09), tricyclic antidepressants (N06AA), selective serotonin reuptake inhibitors (N06AB), antiarrhythmic drugs class I and III (C01B), and digoxin (C01AA05). Other relevant vagal modulating agents like acetylcholinesterase inhibitors and atropine were not represented in the current dataset.

### Statistical analyses

The present work was based on the 4979 CHRIS study participants included in the first data release [21]. After excluding 20 pregnant women and subjects with no information on either HRV or smoking, the available sample size for analysis was of 4751 subjects. Given the relatedness structure of the CHRIS study sample [22], we fitted linear mixed regression models (LMMs) to explore the relationship between HRV indicators and functions of sex, age, and smoking constructs, also allowing for non-linearity and moderating effects. The LMMs’ covariance structure was based on a between-subjects’ relatedness matrix, where pairwise kinship coefficients were estimated from the pedigree using the ‘kinship2’ R package. To reduce skewness and approximate normality, HRV indexes were preliminarily transformed to the natural logarithm, following the Task Force recommendations [1]. After data exploration, all LMMs were finally adjusted for sex, age, age^2^, and sex-by-age^2^ interaction. Never smokers were chosen as the reference category for smoking status in all the analyses. The roles of pack-years and smoking intensity were investigated by means of fractional polynomial (FP) transformations for spike-at-zero variables [28], which supported the inclusion of a linear term for smoking quantity (either pack-years or smoking intensity), while also including smoking status in relevant models. For each outcome separately, the sequence of models included the following terms: (Model A) smoking status; (Model B) smoking status and pack-years as main effects only; (Model C) smoking status and pack-years, plus their interaction; (Model D) smoking status and current smoking intensity, plus their interaction; (Model E) equivalent to Model D, plus the following cardiometabolic risk factors as potential confounders: BMI, hypertension, diabetes, history of cardiovascular events, and physical activity; (Model F) equivalent to Model E, plus use of medication. Statistical significance was set at α = 0.05. All statistical analyses were performed with the R software version 3.3.3 (https://www.r-project.org/). In particular, the LMMs were fitted using the lmekin function in the ‘coxme’ package [29] and the fractional polynomials were explored using the ‘mfp’ package [30] and then integrated in the LMMs.

### Ethics statement

The CHRIS study was approved by the Ethical Committee of the Healthcare System of the Autonomous Province of Bolzano (Südtiroler Sanitätsbetrieb/Azienda Sanitaria dell’Alto Adige), protocol number 21/2011 (19 April 2011). Each participant gave written informed consent.

## Results

Participants had a mean age of 46.2 years (range 18–93), with the youngest age observed for current smokers (**Table 1**), and 55.4% were women. Subjects with hypertension, history of cardiovascular events, and diabetes were 29.0%, 8.9%, and 4.6%, respectively. The study sample included 52.2% never smokers, 29.7% past smokers, and 18.1% current smokers. The mean age at smoking onset was 17.1 years (standard deviation, SD = 3.9), with minimal difference between past (mean = 16.9, SD = 4.3) and current (mean = 17.2, SD = 3.7) smokers. The median smoking duration was 11.0 (interquartile range, IQR: 8.0, 20.0) and 16.5 (IQR: 7.0, 22.3) years for past and current smokers, respectively. Among ever smokers, current and past smokers had smoked a median of 11.2 (IQR: 4.5, 22.0) and 8.5 (IQR: 3.3, 8.5) pack-years, respectively. Among current smokers, the median smoking intensity was 10.0 (IQR: 6.0, 18.0) grams a day. Participants’ characteristics by smoking status are described in **Table 1**.

**Table 1:** Characteristics of the study participants according to smoking status*.

Participants’ characteristics		Never smokers	Past smokers	Current smokers
Sample size (% by row)		2478 (52.2)	1413 (29.7)	860 (18.1)
Demographics	Age, years	47.9 (17.1)	47.9 (14.8)	38.6 (14.5)
	Females	1489 (60.1)	708 (50.1)	438 (50.9)
Cardiometabolic conditions	Hypertension	751 (30.3)	454 (32.1)	170 (19.8)
	History of cardiovascular events	229 (9.2)	145 (10.3)	49 (5.7)
	Diabetes^a^	123 (5.0)	71 (5.0)	22 (2.6)
	BMI^a^, kg/m^2^	25.5 (4.6)	26.1 (4.6)	24.9 (4.2)
Physical activity: IPAQ score^a^	Low	515 (22.2)	334 (24.9)	230 (28.7)
	Medium	672 (28.9)	396 (29.5)	228 (28.5)
	High	1136 (48.9)	614 (45.7)	343 (42.8)
Drugs	No drugs	972 (38.8)	580 (39.8)	386 (44.1)
	HRV-modifying drugs	467 (18.7)	275 (18.9)	75 (8.6)
	Other drugs	1065 (42.5)	601 (41.3)	415 (47.4)
Smoking	Tobacco, g/day		15.0 (8.0, 20.0)	10.0 (6.0, 18.0)
	Smoking Duration, years		11.0 (5.0, 20.0)	16.5 (7.0, 22.3)
	Pack-years		8.50 (3.3, 18.8)	11.2 (4.5, 22.0)
Heart rate	HR, bpm	61.9 (56.21, 67.8)	60.9 (55.7, 66.9)	62.6 (56.9, 68.9)
Heart rate variability (HRV)	SDNN, ms	49.5(37.4, 66.4)	50.8 (38.7, 68.0)	56.3 (40.8, 75.4)
	RMSSD, ms	33.2 (22.9, 50.0)	34.5 (23.2, 51.5)	41.0 (27.1, 61.4)
	TP, ms^2^	1965.1 (1057.8, 3506.9)	2046.3 (1165.4, 3650.9)	2494.1 (1253.2, 4715.5)
	LF, ms^2^	517.2 (256.4, 949.6)	548.8 (284.5, 1008.4)	675.7 (349.6, 1237.5)
	HF, ms^2^	241.4 (106.8, 529.0)	244.8 (112.4, 571.4)	382.61 (143.5, 810.1)
	LF/HF	2.1 (1.3, 3.4)	2.1 (1.3, 3.6)	1.9(1.2, 3.1)

*Reported figures indicate: mean (SD) for age and BMI; median (interquartile range) for tobacco g/day, smoking duration, pack-years, HR, SDNN, RMSSD, TP, LF, HF, and LF/HF; N(%) for females, hypertension, history of cardiovascular events, diabetes, physical activity groups, and drug groups.

^a^ Number of observations may vary slightly, due to missing data.

**Fig 1:**
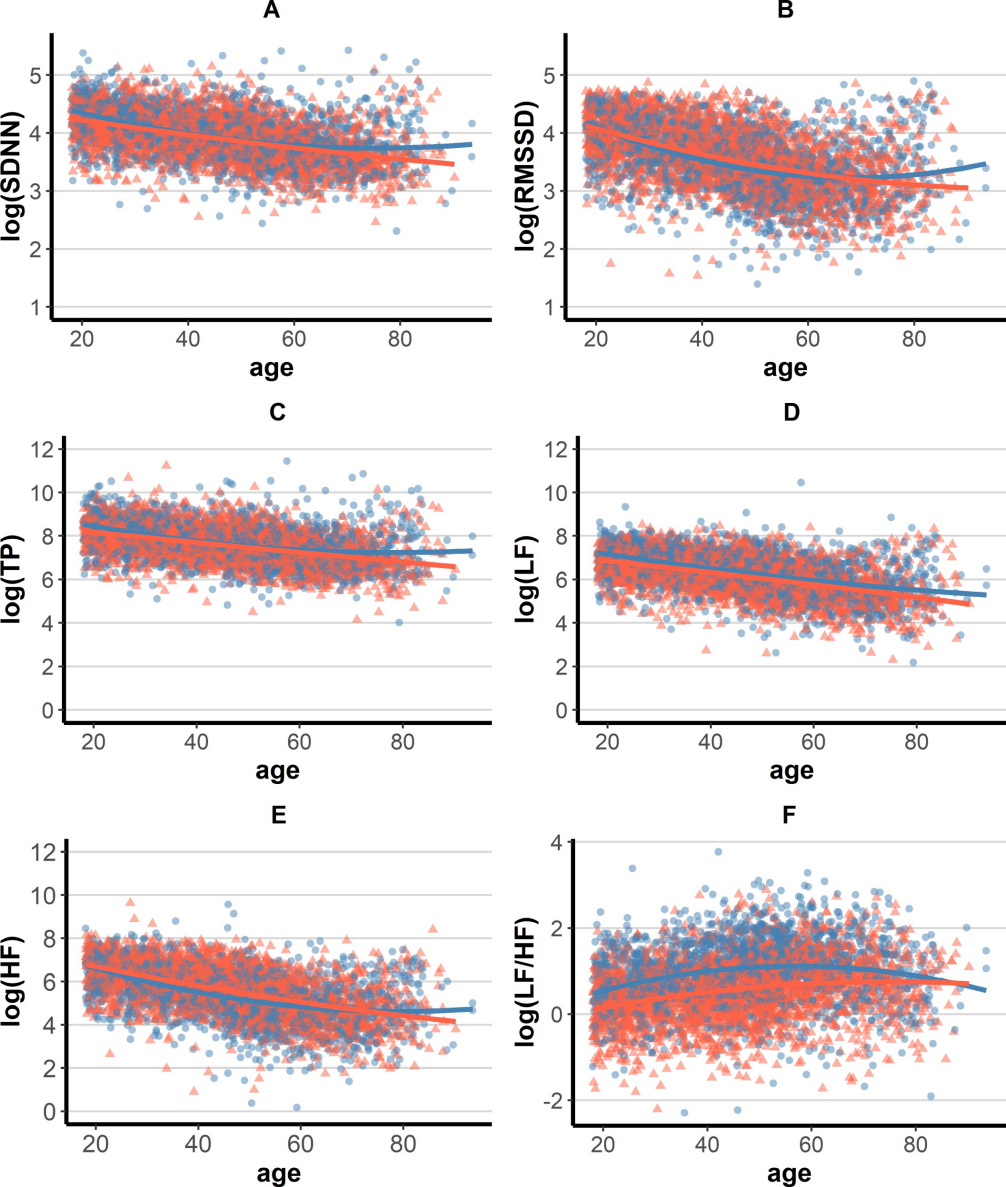
Distribution of HRV indexes by age and sex. Blue: males; red: females. Fitting lines were based on loess quadratic smoothing (**A**) log(SDNN); (**B**) log(RMSSD); (**C**) log(TP); (**D**) log(LF); (**E**) log(HF); and (**F**) log(LF/HF). Measurement units: SDNN and RMSSD are expressed in ms; TP, LF, and HF in ms^2^.

Study participants had median SDNN of 50.8 (IQR: 38.5, 68.7) ms, RMSSD of 34.7 (IQR: 23.4, 52.0) ms, TP of 2075.6 (IQR: 1133.1, 3721.3) ms^2^, LF of 552.5 (IQR: 276.9, 1021.7), HF of 258.7 (IQR: 114.1, 595.9) ms^2^, and LF/HF ratio of 2.1 (IQR: 1.3, 3.4). Log-transformed HRV indexes showed a decreasing linear trend with age (**Fig 1**), corresponding to -0.115 (95%CI: -0.121, -0.108) log(SDNN), -0.172 (95%CI: -0.180, -0.163) log(RMSSD), -0.231 (95%CI: -0.243, -0.217) log(TP), –0.279 (95%CI: -0.294, -0.265) log(LF), -0.387 (95%CI: -0.404, -0.370) log(HF), and 0.104 (95%CI: 0.091, 0.116) log(LF/HF), every 10 years of age. Such a decline with age was also characterized by a quadratic component interacting with the participant’s sex. The HRV decline persisted on a linear trend with age in females, whilst a stabilization and next (approximately after age 60) a trend inversion with age was observed among males (sex × age^2^ interaction *P* value <0.001 for all HRV metrics) (**Table 2**).

**Table 2:** Effect of sex and age^a^ on HRV.

	log(SDNN)		log(RMSSD)		log(TP)		log(LF)		log(HF)		log(LF/HF)	
	Effect (95%CI)	*P* value	Effect (95%CI)	*P* value	Effect (95%CI)	*P* value	Effect (95%CI)	*P* value	Effect (95%CI)	*P* value	Effect (95%CI)	*P* value
Female	-0.009 (-0.036, 0.019)	0.551	0.112 (0.075, 0.149)	<0.001	-0.049 (-0.108, 0.009)	<0.001	-0.172 (-0.234, -0.110)	<0.001	0.321 (0.246, 0.395)	<0.001	-0.481 (-0.535, -0.428)	<0.001
Age	-0.115 (-0.121, -0.108)	<0.001	-0.172 (-0.180, -0.163)	<0.001	-0.231 (-0.243, -0.217)	<0.001	-0.279 (-0.294, -0.265)	<0.001	-0.387 (-0.404, -0.37)	<0.001	0.104 (0.091, 0.116)	<0.001
Age^2^	0.020 (0.014, 0.024)	<0.001	0.038 (0.031, 0.044)	<0.001	0.035 (0.024, 0.045)	<0.001	0.030 (0.005, 0.055)	0.020	0.057 (0.043, 0.070)	<0.001	-0.067 (-0.089, -0.046)	<0.001
Age^2^×Female	-0.017 (-0.023, -0.009)	<0.001	-0.024 (-0.032, -0.014)	<0.001	-0.034 (-0.048, -0.019)	<0.001	-0.017 (-0.032, -0.001)	0.034	-0.042 (-0.06, -0.023)	<0.001	0.024 (0.011, 0.037)	<0.001

Abbreviations: CI: Confidence Interval.

^a^ Age was evaluated by 10-year increases.

**Fig 2:**
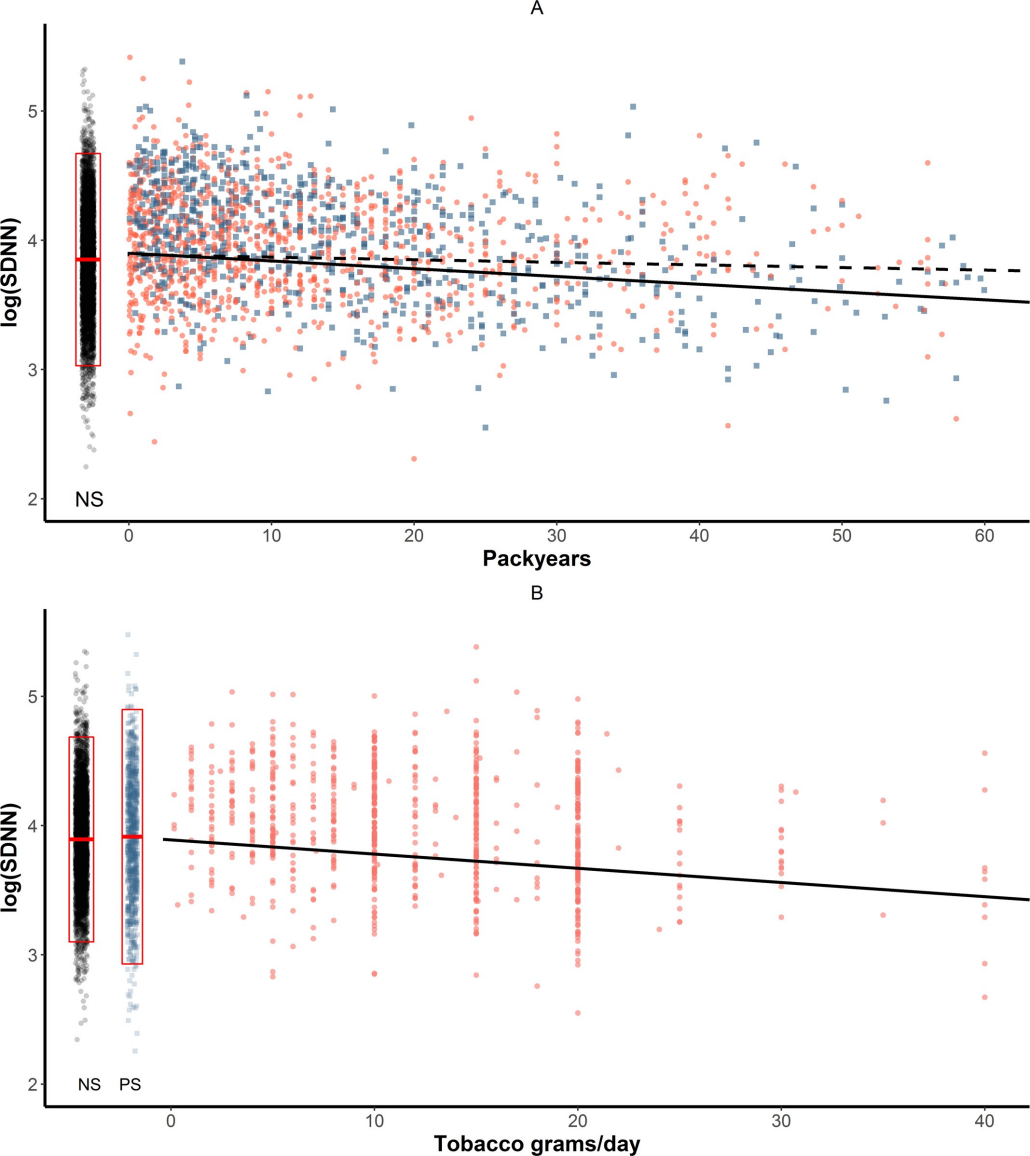
Effect of smoking status, history, and intensity on log(SDNN). (**A**) Never smokers (NS), indicated by black dots in the box-plot, are compared with ever smokers. The box represents the mean log(SDNN) ± 1 SD. Past and current smokers are identified by blue and red dots, respectively. Dashed and solid lines represent the fitted pack-years effect in past and current smokers, respectively, as obtained from *Model C*; (**B**) Never smokers (NS, black dots) and past smokers (PS, blue squares) are compared with current smokers (red dots). Solid line represents the fitted effect of daily tobacco grams on log(SDNN) for current smokers. Measurement units: SDNN is expressed in ms.

Results of the association between smoking and HRV are shown in **Table 3**. In sex- and age-adjusted models, where only smoking status was included as a factor potentially influencing HRV, past smokers showed higher HRV levels than never smokers on all HRV traits (**Table 3**, *model A*). Under this model, no differences were observed between current and never smokers (all *P* values ≥0.16). When additionally taking cumulative smoking into account by adding pack-years information, regression models based on FP functions showed a significant pack-years effect on all HRV metrics of interest (**Table 3**, *model B*). The linear negative effect of each additional 5 pack-years was -0.009 (95%CI: -0.013, -0.004) log(SDNN) and -0.012 (95%CI: -0.021, -0.002) log(RMSSD). Finally, when adding a smoking status-by-pack-years interaction term to the model, we observed that the pack-years effect was almost null among past smokers (effect on log(SDNN) = -0.004, 95%CI: -0.013, 0.005; effect on log(RMSSD) = -0.003, 95%CI: -0.015, 0.009) (**Table 3**, *model C;* **Fig 2**). However, the effect was remarkable among current smokers, where each additional 5 pack-years were associated with -0.020 log(SDNN) (95%CI: -0.033, -0.006) and -0.022 (95%CI: -0.041, -0.004) log(RMSSD). Under this model, at the fictional value of zero pack-years, the projected HRV levels for past smokers and current smokers were higher than that of never smokers; for past versus never smokers, we observed a +0.041 (95%CI: 0.01, 0.07) log(SDNN) and +0.064 (95%CI 0.024, 0.103) log(RMSSD); for current versus never smokers, we observed +0.031 (95%CI:-0.008, 0.070) log(SDNN) and +0.070 (95%CI 0.018, 0.121) log(RMSSD). Analogous results were observed for the frequency domain indicators, with similar effects of smoking on log(LF) and log(HF), resulting in no association with the log(LF/HF) (**Table 3**).

**Table 3:** Association of smoking status and pack-years with HRV^a^.

		Model A		Model B		Model C	
HRV indexes	Exposure variables	Effect (95%CI)	*P* value	Effect (95%CI)	*P* value	Effect (95% CI)	*P* value
**log(SDNN)**	Past Smokers	0.023 (-0.001, 0.048)	0.061	0.029 (0.004, 0.053)	0.019	0.041 (0.011, 0.070)	0.007
	Current Smokers	-0.006 (-0.035, 0.020)	0.710	0.012 (-0.019, 0.043)	0.450	0.031 (-0.008, 0.070)	0.115
	5 PY^b^			-0.009 (-0.013, -0.004)	<0.001		
	5 PY×Past Smokers					-0.004 (-0.013, 0.005)	0.417
	5 PY×Current Smokers					-0.020 (-0.033, -0.006)	0.005
**log(RMSSD)**	Past Smokers	0.047 (0.015, 0.079)	0.004	0.079 (0.040, 0.116)	<0.001	0.064 (0.024, 0.103)	0.002
	Current Smokers	0.028 (-0.011, 0.067)	0.160	0.042 (-0.004, 0.088)	0.077	0.070 (0.018, 0.121)	0.008
	5 PY^b^			-0.012 (-0.021, -0.002)	0.010		
	5 PY×Past Smokers					-0.003 (-0.015, 0.009)	0.656
	5 PY×Current Smokers					-0.022 (-0.041, -0.004)	0.015
**log(TP)**	Past Smokers	0.049 (-0.002, 0.100)	0.061	0.109 (0.049, 0.168)	<0.001	0.077 (0.014, 0.138)	0.016
	Current Smokers	-0.023 (-0.085, 0.039)	0.460	0.003 (-0.070, 0.077)	0.932	0.063 (-0.018, 0.144)	0.130
	5 PY^b^			-0.025 (-0.039, -0.009)	0.001		
	5 PY×Past Smokers					-0.004 (-0.022, 0.015)	0.712
	5 PY×Current Smokers					-0.049 (-0.077, -0.020)	<0.001
**log(LF)**	Past Smokers	0.072 (0.018, 0.126)	0.001	0.080 (0.026, 0.135)	0.004	0.066 (0.011, 0.121)	0.019
	Current Smokers	-0.025 (-0.090, 0.040)	0.455	-0.002 (-0.070, 0.066)	0.961	0.041 (-0.030, 0.113)	0.256
	5 PY^b^			-0.012 (-0.022, -0.001)	0.028		
	5 PY×Past Smokers					-0.006 (-0.008, 0.020)	0.395
	5 PY×Current Smokers					-0.042 (-0.062, -0.021)	<0.001
**log(HF)**	Past Smokers	0.072 (0.006, 0.137)	0.031	0.149 (0.072, 0.225)	<0.001	0.119 (0.039, 0.198)	0.003
	Current Smokers	0.017 (-0.062, 0.096)	0.670	0.041 (-0.052, 0.135)	0.389	0.097 (-0.007, 0.201)	0.068
	5 PY^b^			-0.024 (-0.043, -0.004)	0.013		
	5 PY×Past Smokers					-0.005 (-0.029, 0.020)	0.717
	5 PY×Current Smokers					-0.045 (-0.081, -0.008)	0.015
**log(LF/HF)**	Past Smokers	-0.020 (-0.066, 0.027)	0.405	-0.025 (-0.071, 0.022)	0.305	-0.026 (-0.073, 0.022)	0.290
	Current Smokers	-0.039 (-0.095, 0.017)	0.167	-0.053 (-0.112, 0.006)	0.077	-0.047 (-0.111, 0.012)	0.112
	5 PY^b^			0.007 (-0.002, 0.016)	0.131		
	5 PY×Past Smokers					0.008 (-0.004, 0.020)	0.169
	5 PY×Current Smokers					-0.003 (-0.021, 0.015)	0.733

Abbreviations: PY: pack-years; CI: Confidence Interval.

^a^ All models were adjusted for sex, age, age^2^, and sex×age^2^, and included the smoking variables as they are listed in the table. Never smokers are always the reference category.

^b^ Effects refer to increases of 5 pack-years.

Given pack-years reflect cumulative smoking history but not smoking intensity per se, we replaced the pack-years with tobacco grams a day in current smokers to assess the impact of current smoking intensity on HRV (**Table 4**, *model D*). We observed that each additional 10 grams of tobacco daily smoked corresponded to -0.089 (95%CI: -0.124, -0.054) log(SDNN) (**Fig 2**) and -0.080 (95%CI: -0.126. -0.033) log(RMSSD). At the projected level of zero grams a day, current smokers had higher HRV levels than never smokers: +0.091 (95%CI: 0.038, 0.144) log(SDNN) and +0.114 (95%CI: 0.043,0.183) log(RMSSD). To exclude that the results were influenced by imbalance of cardiometabolic conditions and risk factors between the three smoking status groups, we additionally adjusted for history of cardiovascular events, diabetes, hypertension, BMI, and physical activity (**Table 4**, *model E*). log(SDNN) and log(RMSSD) were associated with BMI, diabetes, and HTN. However, the inclusion of this information did not alter the association between smoking status and smoking intensity with HRV indexes. Results were confirmed when considering the frequency domain indexes (**Table 5**, *model E*). When we additionally took the use of medication into account, we observed no association between using HRV-modifying drugs and HRV indexes. Instead, subjects using other kinds of drugs resulted in lower levels of both time and frequency domain indexes, with the exception of the LF/HF ratio. Nevertheless, including information on medication did not alter the effect of smoking status and smoking intensity on neither the time domain nor the frequency domain HRV indexes (**Table 4** and **Table 5**, *model F*).

**Table 4:** Association of HRV time-domain indexes with smoking status and intensity (model D); smoking status, smoking intensity and cardiometabolic risk factors (model E); and smoking status, smoking intensity, cardiometabolic risk factors, and drugs (model F)^a^.

* *	* *	*Model D*		*Model E*		*Model F*	
HRV indexes	Exposure Variables	Effect (95%CI)	*P* value	Effect (95%CI)	*P* value	Effect (95%CI)	*P* value
log(SDNN)	Past Smokers	0.022 (-0.001, 0.046)	0.071	0.033 (0.008, 0.057)	0.008	0.033 (0.008, 0.058)	0.009
	Current Smokers	0.091 (0.038, 0.144)	<0.001	0.102 (0.048, 0.154)	<0.001	0.101 (0.047, 0.155)	<0.001
	10 Grams/day^b^	-0.089 (-0.124, -0.054)	<0.001	-0.096 (-0.130, -0.060)	<0.001	-0.098 (-0.134, -0.062)	<0.001
	BMI			-0.003 (-0.005, 0.000)	0.015	-0.003 (-0.006, -0.001)	0.016
	HTN (yes vs. no)			-0.054 (-0.104, -0.003)	0.037	-0.055 (-0.107, -0.003)	0.037
	DM (yes vs. no)			-0.068 (-0.123, -0.013)	0.015	-0.069 (-0.124, -0.013)	0.016
	CVD (yes vs. no)			-0.014 (-0.063, 0.034)	0.563	-0.011 (-0.059, 0.040)	0.702
	Physical activity			0.002 (-0.002, 0.005)	0.402	0.002 (-0.002, 0.006)	0.393
	HRV-modifying drugs					-0.022 (-0.062, 0.017)	0.262
	Other drugs					-0.032 (-0.056, -0.007)	0.011
log(RMSSD)	Past Smokers	0.046 (0.013, 0.078)	0.005	0.060 (0.027, 0.092)	<0.001	0.061 (0.027, 0.093)	<0.001
	Current Smokers	0.114 (0.043, 0.183)	0.002	0.122 (0.051, 0.192)	<0.001	0.124 (0.053, 0.195)	0.001
	10 Grams/day^b^	-0.080 (-0.126, -0.033)	<0.001	-0.086 (-0.132, -0.039)	<0.001	-0.089 (-0.136, -0.042)	<0.001
	BMI			0.000 (-0.003, 0.003)	0.877	-0.001 (-0.004, 0.003)	0.686
	HTN (yes vs. no)			-0.094 (-0.160, -0.026)	0.007	-0.106 (-0.175, -0.037)	0.003
	DM (yes vs. no)			-0.118 (-0.191, -0.044)	0.002	-0.122 (-0.196, -0.048)	0.001
	CVD (yes vs. no)			-0.002 (-0.066, 0.063)	0.963	0.003 (-0.062, 0.069)	0.921
	Physical activity			0.002 (-0.003, 0.007)	0.392	0.002 (-0.003, 0.008)	0.415
	HRV-modifying drugs					0.009 (-0.043, 0.061)	0.738
	Other drugs					-0.059 (-0.092, -0.027)	<0.001

Abbreviations: CI: Confidence Interval; HTN: Hypertension; DM: Diabetes; CVD: History of Cardiovascular events.

^a^ All models were adjusted for sex, age, age^2^, and sex×age^2^, and included the smoking variables as they are listed in the table. Never smokers are always the reference category.

^b^ Effects refer to increases of 10 grams/day in current smokers only.

**Table 5:** Association of HRV frequency-domain indexes with smoking status and intensity (model D); smoking status, smoking intensity and cardiometabolic risk factors (model E); and smoking status, smoking intensity, cardiometabolic risk factors, and drugs (model F)^a^.

		Model D		Model E		Model F	
HRV indexes	Exposure variables	Effect (95%CI)	*P* value	Effect (95%CI)	*P* value	Effect (95%CI)	*P* value
log(TP)	Past Smokers	0.048 (-0.003, 0.099)	0.066	0.067 (0.015, 0.118)	0.010	0.066 (0.014, 0.118)	0.013
	Current Smokers	0.171 (0.059, 0.283)	0.003	0.197 (0.085, 0.308)	<0.001	0.196 (0.083, 0.310)	0.001
	10 Grams/day^b^	-0.179 (-0.252, -0.105)	<0.001	-0.195 (-0.268, -0.121)	<0.001	-0.201 (-0.276, -0.126)	<0.001
	BMI			-0.006 (-0.011, 0.000)	0.037	-0.006 (-0.011, -0.000)	0.034
	HTN (yes vs. no)			-0.097 (-0.203, 0.009)	0.075	-0.100 (-0.209, 0.009)	0.072
	DM (yes vs. no)			-0.131 (-0.247, -0.015)	0.026	-0.132 (-0.249, -0.015)	0.028
	CVD (yes vs. no)			-0.034 (-0.136, 0.069)	0.519	-0.027 (-0.131, 0.077)	0.614
	Physical activity			0.004 (-0.005, 0.012)	0.417	0.003 (-0.006, 0.012)	0.467
	HRV-modifying drugs					-0.038 (-0.121, 0.045)	0.369
	Other drugs					-0.066 (-0.118, -0.015)	0.012
log(LF)	Past Smokers	0.071 (0.017, 0.125)	0.011	0.081 (0.027, 0.135)	0.003	0.080 (0.025, 0.135)	0.004
	Current Smokers	0.163 (0.045, 0.281)	0.007	0.170 (0.052, 0.287)	0.005	0.172 (0.053, 0.291)	0.005
	10 Grams/day^b^	-0.168 (-0.246, -0.090)	<0.001	-0.169 (-0.246, -0.091)	<0.001	-0.177 (-0.256, -0.099)	<0.001
	BMI			-0.005 (-0.010, 0.001)	0.104	-0.005 (-0.011, 0.001)	0.086
	HTN (yes vs. no)			-0.055 (-0.167, 0.057)	0.335	-0.059 (-0.173, 0.056)	0.316
	DM (yes vs. no)			-0.075 (-0.197, 0.048)	0.232	-0.073 (-0.196, 0.050)	0.244
	CVD (yes vs. no)			-0.078 (-0.186, 0.030)	0.157	-0.064 (-0.173, 0.046)	0.253
	Physical activity			0.003 (-0.007, 0.012)	0.585	0.002 (-0.007, 0.011)	0.673
	HRV-modifying drugs					-0.072 (-0.158, 0.015)	0.107
	Other drugs					-0.107 (-0.161, -0.053)	<0.001
log(HF)	Past Smokers	0.071 (0.005, 0.136)	0.033	0.096 (0.030, 0.162)	0.004	0.096 (0.029, 0.162)	0.005
	Current Smokers	0.203 (0.060, 0.345)	0.005	0.211 (0.068, 0.354)	0.004	0.222 (0.078, 0.367)	0.003
	10 Grams/day^b^	-0.174 (-0.267, -0.080)	<0.001	-0.180 (-0.274, -0.085)	<0.001	-0.191 (-0.287, -0.096)	<0.001
	BMI			-0.001 (-0.008, 0.005)	0.688	-0.002 (-0.009, 0.005)	0.544
	HTN (yes vs. no)			-0.061 (-0.197, 0.075)	0.379	-0.068 (-0.207, 0.071)	0.335
	DM (yes vs. no)			-0.166 (-0.314, -0.017)	0.029	-0.163 (-0.312, -0.013)	0.033
	CVD (yes vs. no)			-0.112 (-0.243, 0.019)	0.096	-0.109 (-0.242, 0.023)	0.107
	Physical activity			0.003 (-0.008, 0.013)	0.649	0.002 (-0.009, 0.013)	0.728
	HRV-modifying drugs					-0.011 (-0.116, 0.095)	0.845
	Other drugs					-0.094 (-0.160, -0.028)	0.005
log(LF/HF)	Past Smokers	-0.019 (-0.066, 0.027)	0.417	-0.015 (-0.062, 0.032)	0.525	-0.016 (-0.063, 0.032)	0.515
	Current Smokers	-0.047 (-0.148, 0.055)	0.368	-0.042 (-0.144, 0.060)	0.422	-0.050 (-0.153, 0.053)	0.342
	10 Grams/day^b^	0.014 (-0.054, 0.081)	0.693	0.012 (-0.056, 0.079)	0.735	0.014 (-0.054, 0.082)	0.689
	BMI			-0.003 (-0.008, 0.002)	0.192	-0.003 (-0.008, 0.002)	0.258
	HTN (yes vs. no)			0.006 (-0.092, 0.104)	0.904	0.010 (-0.090, 0.109)	0.847
	DM (yes vs. no)			0.091 (-0.015, 0.198)	0.092	0.090 (-0.017, 0.196)	0.100
	CVD (yes vs. no)			0.034 (-0.061, 0.128)	0.485	0.046 (-0.049, 0.140)	0.347
	Physical activity			0.001 (-0.008, 0.008)	0.993	0.001 (-0.008, 0.008)	1.000
	HRV-modifying drugs					-0.061 (-0.136, 0.014)	0.113
	Other drugs					-0.013 (-0.060, 0.035)	0.603

Abbreviations: CI: Confidence Interval; HTN: Hypertension; DM: Diabetes; CVD: History of Cardiovascular events.

^a^ All models were adjusted for sex, age, age^2^, and sex×age^2^, and included the smoking variables as they are listed in the table. Never smokers are always the reference category.

^b^ Effects refer to increases of 10 grams/day in current smokers only.

## Discussion

In this study, we assessed the effects of multiple dimensions of smoking (i.e. smoking status, cumulative smoking history, and smoking intensity) on cardiac autonomic function using HRV metrics in a large general adult population sample. Our results indicate that HRV is strongly associated with smoking intensity, evaluated as grams of tobacco a day, in current smokers. Current smokers exhibited a decaying pattern of HRV level by more than 9% every 10 grams of daily smoked tobacco. Results were confirmed by metrics derived in terms of both time and frequency domains of HRV. Consequently, if smoking intensity is not accounted for, classical smoking status classification among never, past, and current tobacco users, may not entirely reflect the peculiar association with HRV. In our structured analytical framework, the effect due to smoking status became interpretable when we first included pack-years in the model and next, even more so, by the alternative inclusion of current smoking intensity. Further, smoking status and intensity were associated with HRV independently of the most common cardiometabolic conditions, including use of medications that potentially alter HRV levels, suggesting current progressively heavier smoking as an independent risk factor for lower HRV levels. Past smokers exhibited higher levels of HRV than never smokers. This effect was not entirely offset by lower HRV levels per increasing numbers of pack-years.

Our results support recent findings from the SAPALDIA study showing a dose-response effect of smoking on different HRV indicators [15]. Results showing that HRV levels are lower in heavy smokers than in non-smokers were reported by several clinical studies of smaller sample size [31; 32; 33]. Alyan *et al*. noticed that it was not the duration of smoking but the number of cigarettes smoked per day to drive the association with HRV [34]. This supports our findings that HRV is associated with smoking history only among current smokers, while it is also associated with current smoking intensity. The distinctive effect of smoking intensity on HRV might explain why population-based studies are often contradictory concerning the smoking status–HRV association. While some studies show a lower HRV in current [35] or ever smokers [33] as compared to non-smokers, others do not [18; 19; 20]. The inclusion of smoking intensity or, alternatively, history, in addition to smoking status in statistical modeling appears therefore crucial for the dissection of effects due to the different aspects of smoking behavior. Otherwise, if smoking status is considered in isolation, results will depend on the ratio between light and heavy smokers among current smokers, and will vary depending on the specific environmental and cultural context.

Our finding that current heavy smoking is associated with reduced HRV independently of other cardiometabolic risk factors, and both HRV-altering and non-altering drugs, adds evidence to the published literature, which supports analogous associations in very different contexts and groups of participants: healthy old [33] and young [36] individuals, professional workers [32], general population subjects without clinical heart disease [31], and arterial hypertensive patients [37].

Except for LF, which reflects both the sympathetic and parasympathetic systems, all indexes considered in our analysis were mainly indicators of the parasympathetic system activity. Our result show lower levels of all HRV indexes, including LF, at higher smoking intensity levels. By similar effect on LF and HF, smoking intensity had no apparent association with the LF/HF ratio. Overall, these results suggest that the intensity of smoking may have a systemic dysautonomic effect, that is, a direct neurotoxic effect on the whole system, debatable whether central or peripheral. Consistently, regular cigarette consumption has been shown to result in autonomic dysfunction [33]. Of note, most of the effects of smoking on autonomic cardiac regulation have been attributed to nicotine, the main addictive compound of tobacco smoking [38]. The mechanisms of nicotine involve both stimulation and blocking of the autonomic ganglia, release of neurotransmitters, stimulation of the carotid body chemoreceptors and aortic baroreceptors, and direct action to the central nervous system [38]. In particular, nicotine acts on the ANS by activating and desensitizing the peripheral nicotinic acetylcholine receptors (nAChRs), which mediate autonomic ganglionic transmission. However, there are various subtypes of nAChRs and each subtype may implicate a different mediation mechanism at the ganglionic level, with implications on the sympathetic or parasympathetic pathway or both [39]. In addition, in our study, the possible effect of nicotine could not be distinguished from that of other tobacco compounds, such as particulate matter, which may also affect the sympathetic nervous system through different pathways [38]. For these reasons, further considerations on the role of nicotine in the ANS go beyond what can be derive from the analysed data.

Survivor bias may help explaining the complex pattern of associations found in our final analysis, which also includes potential confounders. It is possible that natural selection, especially among the elderly, will operate to preserve those who have better cardiac autonomic function since early ages, independently of lifestyle behaviors. The quadratic relationship with age of HRV decline may in part reflect this pattern. The hypothesized action of natural selection is also compatible with the reversing trend of HRV with age observed among males. Males typically smoke at greater prevalence and higher amounts of tobacco than females at these latitudes [40], hypothetically contributing to lower survival among males and relatively higher HRV compared to women on those surviving. Self-selection into the study, with lower representativeness of the elderly compared to other age groups [21], could also contribute to the age-related trend. Residual confounding may additionally help interpretation. General health conditions and health-prone attitudes may also prompt people never to start, or ever smokers to stop smoking beforehand. For instance, current smokers were the youngest group but also those taking the lowest proportion of HRV-modifying drugs, suggesting a more limited presence of cardiovascular symptoms in this group. Thus, both past smokers and current smokers at ‘virtually’ no current smoking intensity (as reflected by the main effect on current smoking status in Table 4, *model E*) may present similarly higher HRV than never smokers, among the ‘surviving’ participants and independently of major risk factors. However, the observable benefits on HRV may be greater for past smokers than for current smokers, when also accounting for current smoking intake, in accordance with the purportedly acute effect of smoking. For a 10 grams daily increment of current tobacco consumption, the hypothetical benefit on HRV among current smokers of any age and sex would be almost completely nihilated. Consistently with our findings, Harte and colleagues showed that participants who successfully quit smoking, compared to those who did not, exhibited a graded pattern of improvement, characterized by increases in HRV indexes [41]. However, no previous population-based study showed a similar feature. One possible biological interpretation is that improvements in cardiac autonomic function are attributable to nicotine interruption. However, this interpretation is only speculative, given that there was no assessment of plasma nicotine concentrations in our study. The cross-sectional nature of our study and current data availability did not allow investigating these matters further.

To the best of our knowledge, this is the largest cross-sectional study performed to date assessing the relationship between HRV and smoking. Thanks to the large sample size and to the implementation of very detailed smoking behavior questionnaires, we were able to ascertain smoking across multiple dimensions, comprising status, history, and intensity. We estimated smoking effects on HRV by mutually modelling cross products of multiple components of smoking behavior with fractional polynomials for spike-at-zero variables. This has allowed us to observe the conditional impact of each separate component on HRV, in an objective and analytically structured approach. While being logistically unfeasible in our study to submit participants to a 24-hour Holter observation, it was shown that HRV indexes resulting from short-term recordings are strongly correlated with those derived from 24-hour heart rate data and both are equally predictive of mortality after myocardial infarction [42]. One possible limitation related to the 20 minute ECG data is that they might not represent the 24-hour parasympathetic and sympathetic functions and their balance. Previous studies exhibited an influence of the circadian system on the autonomous modulation, with lower HRV levels during daytime than night-time hours [43, 44]. However, all participants in our study performed the ECG at around the same time in the morning under standardized conditions. Following this protocol could have systematically altered their individual HRV levels, but it is unlikely to have aided extra random variability, which would have possibly diluted some associations of interest. A question may arise as to whether a 20 minute recording is better suited for assessing time domain or frequency domain indexes. Following the Task Force recommendations, when investigating short-term recordings of less than two or three minutes, frequency domain indexes should be considered more reliable, while time domain indexes should be preferred in the case of long-term recordings, as the lower stability of heart rate modulations in the longer term makes frequency methods less easily interpretable [1]. Twenty minutes long ECGs such as in our case should be considered a good tradeoff where both time- and frequency-based methods are consistent, as supported by evidence of our results across the different indexes. A limitation of our smoking questionnaires was that they did not allow for discrimination between day- and night-time smoking. In male workers, night-time smoking was suggested to affect cardiac modulation more strongly and acutely than daytime smoking [45]. Therefore, in subjects who had been smoking overnight prior to participation, we might have observed a steeper decline of their HRV levels according to the amount of tobacco consumed, potentially inflating the associations between HRV and current smoking intensity. An additional limitation of our study is that all life-style and most of the clinical information were self-reported, and might thus not fully reflect individuals’ health. For instance, our definition of hypertension based on self-reported doctor diagnosis and a blood pressure measurement taken at a single occasion may not correspond to a correct diagnosis of hypertension. As a consequence, we should be aware that some measurement error may be present in our exposure variables, even though it seems unlikely that it could have biased or further confounded the HRV-smoking relationship discussed here.

Our results should be generalizable to the reference Alpine population where the study was carried out. With the exception of the 75+ year old age group, the age- and sex- distribution of the study sample is consistent with the general population distribution [21]. This is also reflected by the prevalence of current smokers (18.1%), which is very close to the general population estimate in South Tyrol (18.8% in the 11+ year old population) [40]. However, given environmental factors, such as air pollution, may affect HRV in interaction with smoking [38], different results may occur in urban contexts and in regions of different social and cultural background [46].

## Conclusion

In conclusion, current smoking intensity is associated with a steep HRV reduction, as highlighted by both time and frequency domain indexes. Different components of smoking behavior may affect HRV in different ways. Sole inclusion of any single component may lead to erroneous conclusions. We warrant longitudinal studies to adopt our comprehensive modelling strategy, encompassing active smoking status, history, and intensity, with possible extension to passive smoking exposure, to illuminate further the smoking-HRV relationship on a temporal scale.
